# Cellular effects of a turmeric root and rosemary leaf extract on canine neoplastic cell lines

**DOI:** 10.1186/s12917-017-1302-2

**Published:** 2017-12-13

**Authors:** Corri B. Levine, Julie Bayle, Vincent Biourge, Joseph J. Wakshlag

**Affiliations:** 1 Department of Clinical Sciences,Veterinary Medical Center C2-009, Cornell University College of Veterinary Medicine, Ithaca, NY 14853 USA; 2 Royal Canin Research Center, Airmargues, France

**Keywords:** Apoptosis, Canine cancer, Mammary carcinoma, Osteosarcoma, Mastocytoma, Curcumin, Rosemary

## Abstract

**Background:**

The use of nutraceuticals is gaining in popularity in human and canine oncology with a relatively limited understanding of the effects in the vastly different tumor types seen in canine oncology. We have previously shown that turmeric root (TE) and rosemary leaf (RE) extracts can work synergistically to reduce neoplastic cell growth, but the mechanisms are poorly understood and require further elucidation.

**Results:**

Three different canine cell lines (C2 mastocytoma, and CMT-12 mammary carcinoma, D17 osteosarcoma) were treated with 6.3 μg mL^−1^ extract individually, or 3.1 μg mL^−1^ of each extract in combination based on studies showing synergy of these two extracts. Apoptosis, antioxidant effects, cellular accumulation of curcumin, and perturbation of signaling pathways were assessed. The TE + RE combination treatment resulted in Caspase 3/7 activation and apoptosis in all cell lines, beyond the effects of TE alone with the CMT-12 cell line being most susceptible. Both extracts had antioxidant effects with RE reducing reactive oxygen species (ROS) by 40–50% and TE reducing ROS by 80–90%. In addition RE treatment enhanced the c-jun N-terminal kinase (JNK) activity in the C2 cell line and TE + RE exposure increased activated JNK by 4–5 times in the CMT-12 cell line. Upon further examination, it was found that RE treatment caused a significant increase in the cellular accumulation of curcumin by approximately 30% in the C2 and D17 cell lines, and by 4.8-fold in the CMT-12 cell line. This increase in intracellular curcumin levels may play a role in the synergy exhibited when using TE and RE in combination.

**Conclusions:**

The use of RE in combination with TE induces a synergistic response to induce apoptosis which is better than either extract alone. This appears to be related to a variable increased TE uptake in cells and activation of pathways involved in the apoptotic response.

## Background

The use of natural remedies, or nutraceuticals, in the treatment of cancer and a variety of other diseases appears prevalent in human and veterinary medicine. The use of plant extracts has been around for centuries, but investigations into the mechanisms of action across various cancer cell lines are more recent, and appear to be highly variable in cell culture systems [1–3]. The effective compounds of interest have been purified from a variety of plants and are used in treating various diseases, including cancer [4]. The benefit of using these plant extracts to treat cancer is the potential synergy of multiple compounds found within a single extract whereby the major compound may have one or more targets, while other molecules in the extract may be affecting other targets or influencing absorption kinetics [5].

The effects of these purified compounds have been examined in vitro in a variety of human cell lines derived from tumors of the colon, skin, and breast tissue [6], but only a few studies have looked at the effects in canine cancer cells lines [7–9]. The major types of cancer found in the dog differ from humans with lymphoma, mast cell disease, osteosarcoma, and mammary neoplasia being most often diagnosed in canine oncology [10]. We previously identified two extracts, turmeric extract rich in curcuminoids (TE) and rosemary leaf extract rich in carnosic acid (RE), which were shown to be cytotoxic and reduce proliferation in a synergistic manner in canine mastocytoma, mammary carcinoma, and osteosarcoma cell lines [11]. The use of TE and its major compound of interest, curcumin, has been extensively studied to treat a variety of diseases and ailments, perhaps due to its ability to bind and interact with a variety of cellular proteins [12]. Unfortunately, the use of TE in vivo has been limited by its poor bioavailability and efforts are still underway to increase the absorption and bioavailability of the curcuminoids found in this extract [13]. This obstacle may be overcome through the use of combination treatments with other extracts that improve bioavailability or hinder additional pathways [14–16]. In our previous study, RE worked in a synergistic manner with TE to decrease cellular proliferation when used in combination. Carnosic acid, the compound of interest in RE, can target a variety of signaling pathways, many of which overlap with those targeted by curcumin. The effects of these two compounds in combination have been examined in acute myeloid leukemia cells and breast cancer cells [17, 18], showing synergy in anti-proliferative effects and increased pro-apoptotic signaling. The safety of these commonly used feed ingredients and continual synergy between the extracts make them candidates for inclusion in the diet as a potential adjuvant treatment for dogs diagnosed with neoplasia, if appropriate serum concentrations can be achieved.

The objective of this in vitro study was to determine the effects on canine cancer cell death and possible mechanisms by which TE and RE exert anti-proliferative and cytotoxic effects individually and in combination on canine mastocytoma, mammary carcinoma, and osteosarcoma cell lines. Concentrations were chosen based on our prior publication surrounding the effective concentrations for synergy between the two extracts of interest [11]. Markers of apoptosis, antioxidant capabilities, and changes in the activation of common cell signaling pathways were analyzed after treatment.

## Methods

### Natural extracts

Turmeric extract (TE; 88% total curcuminoids, #DA251471 Naturex, Avignon, France) and rosemary extract (RE; 67% carnosic acid, #302036 Vitiva, Markovcih, Slovenia) were solubilized in 100% dimethyl sulfoxide (DMSO; Sigma-Aldrich, St. Louis, MO, USA) at 20 mg mL^−1^. Fresh extract stock solutions were prepared and used for every experiment.

### Cell culture

Three canine neoplastic established cell lines, representing hematopoietic, epithelial, and mesenchymal tumor types were used for all experiments; mastocytoma C2 (Dr. Warren Gold, University of California, San Francisco, USA), mammary gland carcinoma CMT-12 (Dr. R. Curtis Bird, Auburn University, Alabama, USA), and osteosarcoma D17 (#CCL-183; ATCC, Manassas, VA, USA). These cell lines were chosen for initial screening as representative cell lines of the three major cell lineages of cancer in dogs in hopes of finding a similar global effect across different cell lineages. Cell lines were grown on tissue culture-treated plates (Laboratory Product Sales, Rochester, NY, USA) at 37 °C and 5% CO_2_ for all experiments and passage of cells, unless otherwise noted. Cell lines were cultured in appropriate complete medium as previously described.^11^ All culture reagents were purchased from Invitrogen, Carlsbad, CA, USA, unless otherwise indicated.

### Apoptosis–associated caspase 3/7 activation assay

Cells were plated at a density of 4 × 10^3^ cells per well on white walled 96-well tissue culture-treated plates (ThermoFisher Scientific, Waltham, MA, USA) and incubated overnight in complete medium. Cells were treated the following day with DMSO vehicle control, 6.3 μg mL^−1^ extract alone, or 3.1 μg mL^−1^ each extract in combination for 36 h. Chemotherapeutic drugs at a 50% inhibitory concentration (IC_50_) were used as a positive control; 12.5 nM toceranib phosphate (Palladia™, Zoetis Animal Health, Florham Park, NJ) was used for the C2 cell line, and 0.3 or 0.5 μM doxorubicin hydrochloride (Sigma Aldrich, St Louis, MO) was used for the CMT-12 and D17 cell lines, respectively. Background fluorescence and luminescence was measured in wells containing treatments but no cells. Caspase 3/7 activation was quantified using the ApoLive-Glo™ Multiplex Assay (Promega, Madison, WI, USA) following manufacturer’s instructions. Briefly, after 36 h of treatment, viability reagent was added to the wells and incubated at 37 °C for 30 m and fluorescence was measured at 400_Ex_/505_Em_. Next, Caspase-Glo 3/7 Reagent was added to all wells, incubated for 30 m at room temperature, and luminescence was measured. Fluorescence and luminescence was measured using SpectraMax M3 Microplate Reader (Molecular Devices, Sunnyvale, CA, USA).

### Flow Cytometry

Cells were plated on 60 mm tissue culture-treated plates (LPS, Rochester, NY) and incubated in complete medium until 60% confluent. Cells were then treated with medium, DMSO vehicle control, extract alone, or extracts in combination. Cells were treated for 12 h (reactive oxygen species generation), 24 h (curcumin accumulation), or 48 h (Apoptosis/Necrosis, Cell Cycle). All flow cytometric analysis was performed on BD FACSCalibur (BD Biosciences, San Jose, CA, USA).

### Cell cycle analysis

Cell cycle effects were analyzed after 24 h (data not shown) and 48 h treatment using propidium iodide staining to label DNA content. Briefly, cells were detached with Accumax cell dissociation solution (Innovative Cell Technologies, San Diego, CA USA), collected in tubes with 1% fetal bovine serum (FBS) in Phosphate Buffered Saline (PBS) and centrifuged for 5 m at 300 rcf at 4 °C. The cell pellet was washed twice with 1% FBS in PBS, filtered, and resuspended in 70% cold ethanol for overnight fixation. The following day, samples were centrifuged for 10 m at 500 rcf at 4 °C, resuspended in cold PBS. Samples were centrifuged again for 5 m at 300 rcf at 4 °C and resuspended in DNA staining solution [2% propidium iodide (Sigma Aldrich), 0.1% Triton X-100 (Sigma Aldrich), in PBS]. Samples were then incubated for 30 m at room temperature and analyzed with an excitation of wavelength of 488 nm and emission of 617 nm. Only C2 and D17 cell lines were analyzed due to the presence of frequent doublets with CMT-12 cells resulting in an artificial accumulation in the G2/M phase.

### Apoptosis and necrosis assay

Apoptosis and necrosis was measured after 48 h treatment using Annexin-V and 7-AAD staining. Briefly, cells were detached with Accumax dissociation solutions (Innovative Cell Technologies, San Diego, CA, USA), collected and centrifuged for 10 m at 500 rcf at 4 °C. The cell pellet was washed once with PBS before resuspension in Annexin Binding Buffer (ABB; 10 mM HEPES, 140 mM NaCl, 2.5 mM CaCl_2_, pH 7.4) at a density of 1 × 10^6^ cell mL^−1^. Annexin-V 488 conjugate and 7-Aminoactinomycin D (7-AAD) were added to the cell suspensions and incubated for 15 m at room temperature. After the incubation, ABB was added to the cell suspension and kept on ice until fluorescence analysis. Events labeled only Annexin-V positive were considered to represent apoptotic cells; events labeled Annexin-V positive and 7-AAD positive were considered to represent necrotic cells.

### Intracellular reactive oxygen species (ROS) analysis

Since the main constituents of TE and RE (curcumin and carnosic acid, respectively) have been implicated as antioxidants, Dihydrorhodamine123 (DHR123; Invitrogen, Carlsbad, CA, USA) assay was used to determine the amount of reactive oxygen species (ROS) present after 12 h treatment with each extract according to literature [19]. Briefly, cells were detached using Accumax dissociation solution (Innovative Cell Technologies), collected and centrifuged for 10 m at 500 rcf at 4 °C. The pellet was washed once with PBS before resuspension in 1 mL of stain (30 μM DHR123 in DMEM). The cell suspension was then incubated at 37 °C for 30 m, pelleted, and resuspended in 1 mL DMEM and filtered before fluorescence analysis of cells.

### Cellular accumulation of curcumin

The cellular accumulation of curcumin was measured by exploiting the auto-fluorescent properties of this compound [20]. After 24 h treatment, cells were detached with Accumax dissociation solution (Innovative Cell Technologies), collected and centrifuged for 10 m at 500 rcf at 4 °C. The cell pellet was washed once with PBS before resuspension in DMEM, and filtered before fluorescence analysis when excited at a wavelength of 488 nm and then measuring emission using a 530/30 filter.

### Western blotting assessment of affected signaling pathways

Cells were plated on 100 mm tissue culture-treated plates (LPS) and incubated overnight in complete medium until 60% confluency was reached. Cells were treated the following day with DMSO vehicle control, 6.3 μg mL^−1^ extract alone, or 3.1 μg mL^−1^ each extract in combination. Cells were harvested and lysed at 12 h and 24 h after treatment using Mammalian Lysis Buffer (MLB; 25 mM Tris, 100 mM NaCL, 1 mM EDTA, 1% Triton X-100, 0.004% NaF, 1 mM NaVO4, 25 mM -glycerophosphoric acid, 100 μg/ml phenylmethanesulfonyl fluoride, and 1 μg/ml each aprotinin and leupeptin, pH 7.4) and sonication, and then centrifuged for 5 m at 14,000 rcf at 4 °C. The supernatant was collected and the protein concentration was determined using the Bradford assay (Coomassie-dye; ThermoFisher Scientific Pierce, Waltham, MA, USA). Samples were equilibrated to a common volume (μg μL^−1^) in MLB and 5× laemmili loading buffer (300 mM Tris-HCl pH 6.8, 10% Sodium dodecyl sulfate, 50% glycerol, 12.5% β-Mercaptoethanol, 0.025% Bromophenol blue). For each protein of interest, 30 μg total proteins were subjected to sodium dodecyl sulfate polyacrylamide gel electrophoresis (SDS-PAGE) on gels ranging from 6 to 15% based on the molecular weight of the protein of interest. The proteins were then transferred to 0.45 μm pore size polyvinylidene fluoride membrane (Immobilon-P Membrane; EMD Millipore, Billerica, MA, USA) for 1 h at 333 mA and then blocked in 5% milk in Tris-buffered saline/0.05% Tween-20 solution (TBST). Membranes were incubated overnight in primary antibody solutions at a dilution of 1:1000 in TBST on a rocking platform at 4 °C. Primary antibodies included mouse anti- phosphorylated-gamma H2A.X and extracellular regulated kinase (ERK) (R&D Biosciences, Boston, MA, USA); mouse anti- Thr202/Tyr204 phosphorylated p44/42 MAPK (ERK1/2) and STAT3 (Cell Signaling Technology, Danvers, MA, USA); rabbit anti- protein kinase B (AKT), Ser473 phosphorylated-AKT, stress-activated protein kinase/jun-N-terminal kinase (SAPK/JNK), Thr183/Tyr185 phosphorylated-SAPK/JNK, focal adhesion kinase (FAK), Tyr397 phosphorylated-FAK, Tyr576/Tyr577 phosphorylated-FAK, Tyr925 phosphorylated-FAK, Src, Tyr416 phosphorylated-Src, Tyr527 phosphorylated-Src, mammalian target of rapamycin (mTOR), Ser2448 phosphorylated-mTOR, Janus kinase 2 (JAK2), Tyr1007/Tyr1008 phosphorylated-JAK2, Ser727 phosphorylated-signal transducer and activator of transcription 3 (STAT3), Tyr705 phosphorylated-STAT3, B-Cell CLL/Lymphoma 2 (BCL2), and BCL2-Associated X Protein (BAX) (Cell Signaling Technology). Membranes were washed three times with TBST and incubated at room temperature for 1 h in the corresponding secondary anti-mouse IgG or anti-rabbit IgG horseradish peroxidase-conjugated antibody at a dilution of 1:2000 (Cell Signaling Technology). Membranes were washed three times with TBST and visualized with a chemi-luminescent reagent (Clarity Western ECL Substrate; Bio-Rad, Hercules, CA, USA). Digital images were captured using an imaging system (Biospectrum 410; UVP, Upland, CA, USA). After images were collected, membranes were washed three times in TBST and incubated with a 1:10,000 dilution in TBST of the house-keeping antibody β-Actin (Sigma-Aldrich) for 1 h at room temperature. Membranes were washed, incubated with mouse secondary antibody at a dilution of 1:2000, and imaged as described.

### Data management and statistical analysis

For all flow cytometry experiments, 10,000 events were collected per sample and then gated based on a forward-scatter/side-scatter plot. Minimally three independent experiments were examined for each treatment through the different assays (percent of gated cells in each phase cycle, percent of apoptotic cells, intracellular ROS and curcumin level) and analyzed with Cell Quest software (BD Biosciences). DMSO was compared against cells in media alone showing no significant differences, therefore DMSO treated cells were used as the control sample for all comparisons. The geometric mean fluorescence (GMF) from each treatment was compared to the DMSO treated samples and represented as fold change for all experiments using GMF due to the differences in fluorescence intensity across cell lines. In addition, for measurements of ROS, an unstained control was used to determine the baseline GMF of each extract. This value was subtracted from the GMF of stained samples to correct for any shift due to auto-fluorescence of the extract with cells alone. Caspase 3/7 activation was determined as caspase activation per total viable cells for each treatment. Raw data from the viability portion of the assay (individual fluorescence values of each well) were normalized to the vehicle alone treatment for each cell line, considered to represent 100% proliferating cells. The ratio of caspase activation to viable cells is represented as fold increase over DMSO treatment alone. Each of the treatment conditions were completed in duplicate and averaged in four independent experiments. Western blots were run in three independent time course experiments and densitometry was completed using ImageJ [21]. Values are represented as a ratio of phosphorylated protein to total protein and standardized to DMSO vehicle control at every time point examined.

All statistical analyses were performed using JMP Pro (v. 11.2.1; SAS Institute Inc., Cary, NC, USA). The residuals of all statistical models were found to be normally distributed therefore parametric statistics were utilized. The fold-change data from caspase 3/7 activation, percent of apoptotic cells, intracellular ROS level and curcumin accumulation assays and the ratio data from western blot assay were processed using analysis of variance with Tukey’s method for multiple comparisons between all treatment conditions (single, combination and DMSO control). In the case of cell cycle dynamics, Dunnett’s method was used to control for multiple comparisons when studying the percent of gated events difference between single treatment or dual combination and DMSO control only at each time point. Differences were considered statistically significant at *p* < 0.05.

## Results

### Turmeric and rosemary extracts effects on cell cycle

**Fig. 1:**
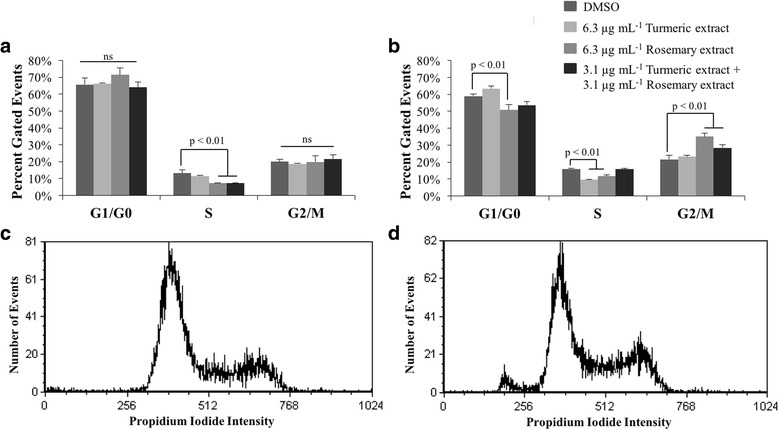
Effects of turmeric and rosemary extract on cell cycle in C2 and D17 cell lines. Cells were treated with indicated concentrations of extracts or DMSO for 48 h and the DNA contents were analyzed using propidium iodide staining by flow cytometry. Percentages of cells within each cell cycle phase (G1, S, and G2/M) were expressed as mean ± standard deviation in (**a**) C2 and (**b**) D17 cell lines. Bar graphs represent the average of three individual experiments performed in duplicate and representative cell cycle histograms of DMSO control treated (**c**) C2 and (**d**) D17 cell lines are shown at 48 h of treatment. All treatments were compared to DMSO vehicle control. Treatments which induced a statistically significant change from DMSO within each cell cycle phase are indicated

The effects of TE and/or RE on cell cycle progression were measured on C2 and D17 tumor cell lines using propidium iodide staining. Cell cycle dynamics were analyzed after 24 h and 48 h of incubation with the different treatments; no significant difference was seen between these two time-points therefore only data from the 48 h time point is shown (Fig. 1A-B). Representative histograms are shown for C2 (Fig. 1C) and D17 (Fig. 1D) cell lines. Single treatment with 6.3 μg mL^−1^ TE resulted in a significant decrease in S phase (DNA replication) in the D17 cell line compared to DMSO control. Treatment with 6.3 μg mL^−1^ RE induced a significant decrease in G_1_/G_0_ phase in the D17 cell line, a reduction in S phase in both cell lines, and an increase in G_2_/M phase (cell division) in the D17 cell line. The combination treatment using 3.1 μg mL^−1^ both extracts induced a small decrease in S phase in only the C2 cell line, and a modest increase in G_2_/M phase in only the D17 cell line. While these differences were significant, the mild alterations in cell cycle in the D17 and C2 of 5–10% decrease in the G_1_/G_0_ and increase in G_2_/M phases with RE and less consistent yet similar changes with both TE and RE combined were not considered large enough to continue examining pathways related to cell cycle arrest as observed in prior studies using these cell lines [22].

### Cellular apoptosis is induced by turmeric and rosemary extract treatments.

**Fig. 2:**
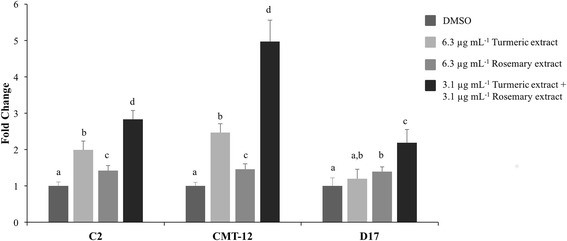
Caspase 3/7 activation induced by turmeric and rosemary extracts in C2, CMT-12, and D17 cell lines. Cells were treated with indicated concentrations of extracts or DMSO for 36 h. Activated caspase 3/7 per viable cells was expressed as mean fold change from DMSO control values ± standard deviation from three independent replicates. Within each cell line, values with different letters are significantly different from each other (C2 *p* < 0.001; CMT-12 *p* < 0.005; D17 *p* < 0.05)

**Fig. 3:**
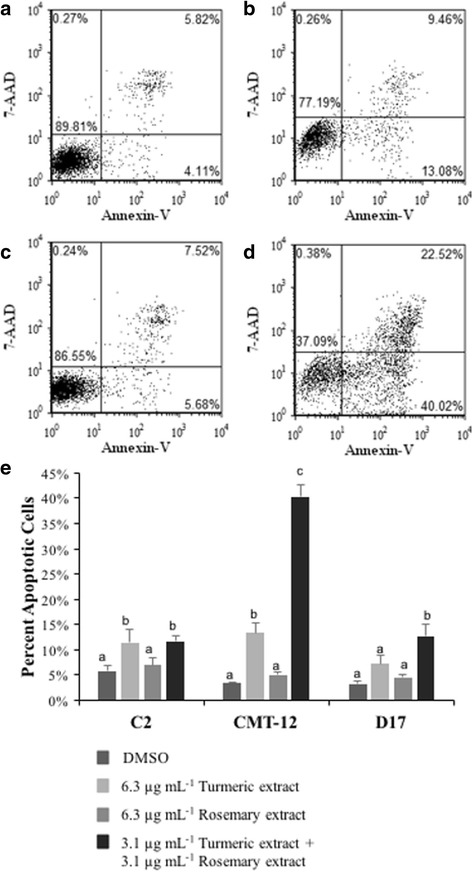
Apoptosis induction by turmeric and rosemary extracts in C2, CMT-12, and D17 cell lines. Cells were incubated with the indicated treatments for 48 h and the induction of apoptosis was detected by Annexin V-FITC and 7-AAD staining followed by flow cytometric analysis. Representative quadrant plots of the CMT-12 cell line treated with (**a**) DMSO, (**b**) 6.3 μg mL^−1^ TE, (**c**) 6.3 μg mL^−1^ RE, or (**d**) 3.1 μg mL^−1^ TE + 3.1 μg mL^−1^ RE are shown. Each quadrant represents the number of events considered live (lower left), early apoptotic (lower right), or late apoptotic/necrotic (upper right). **e** Percent early apoptotic cells (lower right quadrant of Annexin V positive and 7-AAD negative cells) are represented as mean ± standard deviation (three independent replicates). Within each cell line, means with different letters are significantly different from each other (*p* < 0.05)

After treating cells for 36 h, TE alone (6.3 μg mL^−1^) resulted in a significant increase in apoptotic cells in the C2 and CMT-12 cell lines as determined by Caspase 3/7 activation, 2- and 2.5-fold, respectively (Fig. 2), and Annexin-V staining which increased from 6% to 11% apoptotic cells in the C2 (Fig. 3E) and from 4% to 13% in the CMT-12 cell line (represented in Fig. 3A and C lower right quadrant; full analysis Fig. 3E). A treatment with 6.3 μg mL^−1^ RE alone resulted in a statistically significant increase of 1.4-fold in Caspase 3/7 activation in all three cell lines when compared to vehicle control. When the dual combination treatment (3.1 μg mL^−1^ TE + 3.1 μg mL^−1^ RE) was used, a significant increase in Annexin-V positive cells compared to vehicle control was seen in the 3 cell lines, but this was not significant compared to 6.3 μg mL^−1^ TE alone in C2 cell line. However, in the CMT-12 and D17 cell lines, the combination treatment induced a significantly greater percentage of apoptotic cells, 40% and 13%, respectively, compared to 6.3 μg mL^−1^ TE alone (13% and 7%) and 6.3 μg mL^−1^ RE alone (5%) (Fig. 3). This was further validated with the caspase activation assay in which all three cell lines (C2, CMT-12 and D17) showed a significant increase in cleaved Caspase 3/7 when the combination treatment was used (2.8, 5- and 2.2-fold, respectively), compared to TE alone (2-, 2.5- and 1.2-fold) or RE alone (1.4-fold across all three cell lines).

### Antioxidant activity of TE and RE in cancer cell lines

**Fig. 4:**
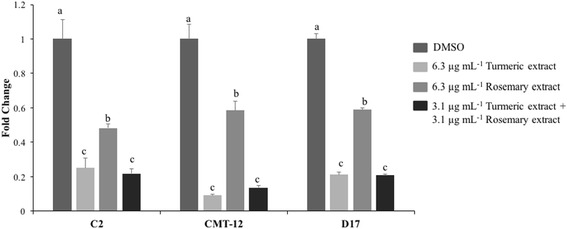
Antioxidant effects of turmeric and rosemary extracts in C2, CMT-12 and D17 cell lines. Cells were treated with the indicated concentrations of extracts for 12 h followed by determination of intracellular levels of reactive oxygen species using Dihydrorhodamine123 staining. Values are expressed as mean ± standard deviation of four independent replicates. Reported values are represented as fold change compared to DMSO vehicle control. Within each cell line, means with different letters are significantly different from each other (C2 p < 0.05; CMT-12 and D17 *p* < 0.0001)

TE alone was a significantly stronger antioxidant than RE alone using same extract concentration (6.3 μg mL^−1^) in all the three cell lines (C2, CMT-12 and D17) with TE reducing ROS by about 75–90-80%, respectively and RE reducing ROS by about 50–40-40%, respectively. The dual combination treatment using half the concentration (3.1 μg mL^−1^ each extract) was as effective as 6.3 μg mL^−1^ TE alone in all three cancer cell lines (Fig. 4).

### Cellular accumulation of curcumin induced by RE treatment

**Fig. 5:**
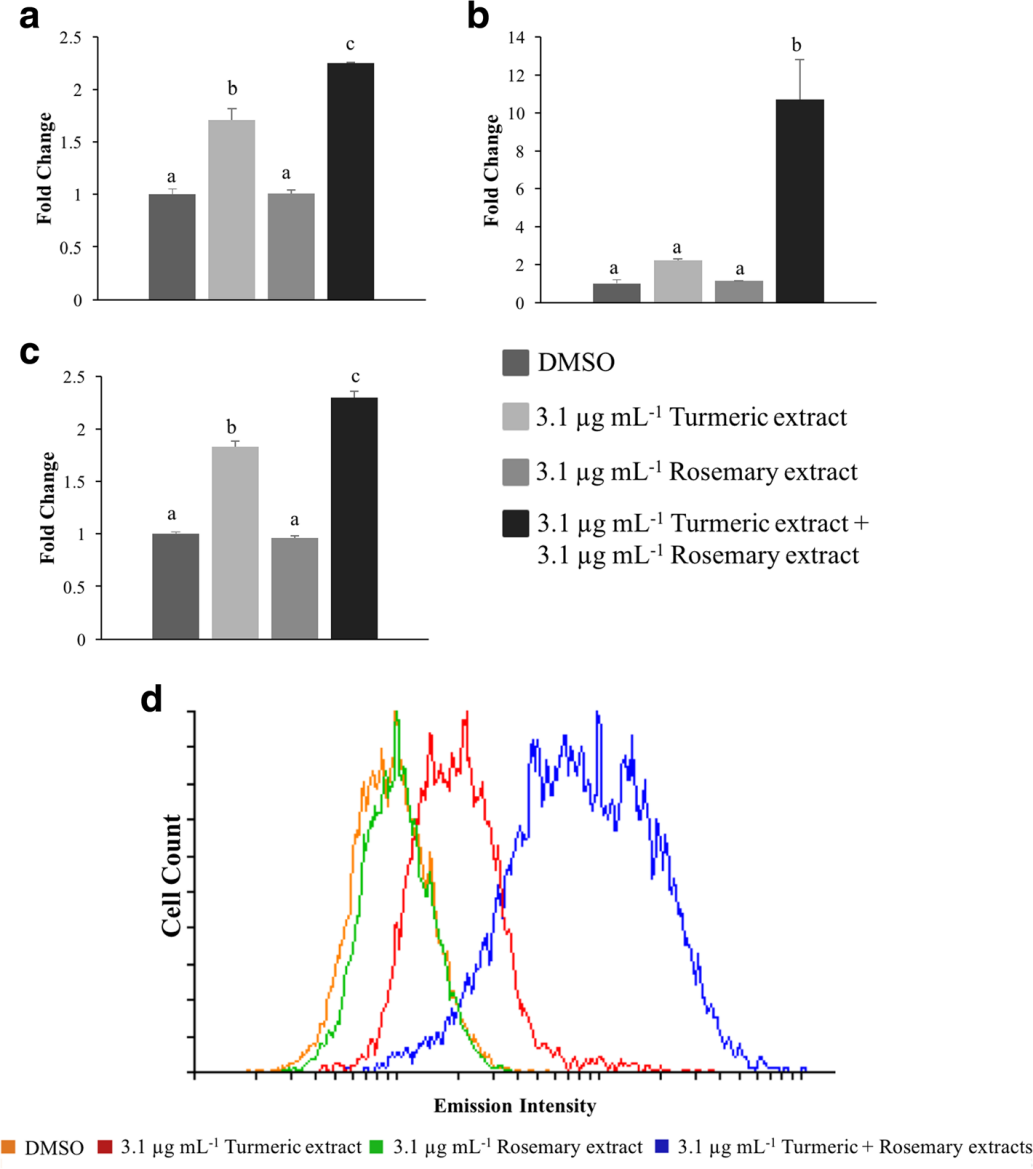
Effect of rosemary extract on intracellular accumulation of curcumin in canine tumor cell lines. The C2 (**a**), CMT-12 (**b**), and D17 (**c**) cell lines were treated with the indicated concentration of extracts for 24 h and then cellular accumulation of curcumin was quantified by flow cytometry. Y-axis values represent the fold change in geometric mean fluorescence (GMF) of all cells compared to DMSO control. Reported data are expressed as mean ± standard deviation of 4 independent replicates. Within each cell line, means with different letters are significantly different from each other (*p* < 0.0001). **d** Representative histogram of emission intensity in CMT-12 cell line after 24 h treatment with DMSO, 3.1 μg mL^−1^ TE alone, 3.1 μg mL^−1^ RE alone, or 3.1 μg mL^−1^ TE + RE combination is shown

Observation from previous flow cytometry experiments showed an unexpected increase in the GMF when cells were treated with TE alone when excited at a wavelength of 488 nm, whereas no change was observed when RE was used alone (Fig. 5D). A similar increase in GMF was also seen when half the concentration of each extract was used in combination (data not shown). Therefore, the possibility that RE could increase the cellular accumulation of the fluorescent compound curcumin was investigated when these compounds were used in combination. TE alone (3.1 μg mL^−1^) significantly increased the GMF in the C2 and D17 cell lines, 1.7- and 1.8-fold, respectively; while when using RE with TE the increase was 2.2 and 2.3 fold, respectively (Fig. 5A, C; *p* < 0.0001). The addition of RE at the same concentration to TE resulted in a significant increase in GMF of 4.8-fold in the CMT-12 cell line beyond that of TE alone (Fig. 5B; *p* < 0.0001).

### TE and RE SAPK/JNK activation

**Fig. 6:**
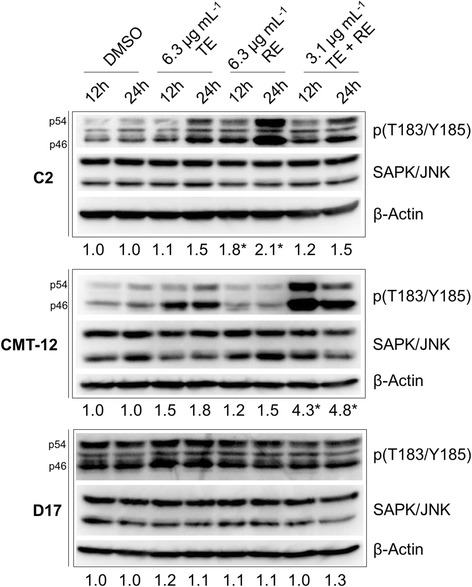
Changes in the protein expression levels of SAPK/JNK pathway in turmeric and rosemary-treated cells. C2, CMT-12 and D17 cell lines were harvested and lysed after 12 h or 24 h treatment with DMSO vehicle control, or 6.3 μg mL^−1^ Turmeric extract (TE) alone, or 6.3 μg mL^−1^ Rosemary extract (RE) alone, or combination of 3.1 μg mL^−1^ each of TE + RE. Expression level of Thr183/Tyr185 phosphorylated-SAPK/JNK (p46/p54) and total SAPK/JNK were determined by Western blot analysis. Each blot is a representative of three independent experiments. Densitometry values represent a ratio of phosphorylated protein to total protein and normalized to DMSO vehicle control of the same time point (mean of three separate experiments). Changes in densitometry compared to DMSO control with significance of *p* < 0.05 represented by *. β-Actin was used as a loading control for every blot to ensure even loading of samples

After examination of several cell signaling pathways, no consistent trend was seen in the phosphorylation status of the variety of signaling proteins, alterations in the mitochondrial proteins involved in apoptosis or markers of DNA damage (data not shown). However, changes in Thr183/Tyr185 phosphorylated-SAPK/JNK (p-SAPK/JNK; Fig. 6) were detected in the three cancer cell lines. Treatment with 6.3 μg mL^−1^ TE resulted in an increase from a densitometry value of 1.1 at 12 h to 1.5 at 24 h in p-SAPK/JNK in the C2 cell line, stable activation from 12 h to 24 h in the CMT-12 cell line (1.5 and 1.8, respectively). In the D17 cell line only a minor non-significant increase was observed (1.2 at 12 h, 1.1 at 24 h). Activated SAPK/JNK increased from 12 h to 24 h in the C2 cell line (1.8 and 2.1, respectively) and the CMT-12 cell line (1.2 at 12 h to 1.5 at 24 h) which was not significant over time. Minimal change was seen in the D17 cell lines after treatment with 6.3 μg mL^−1^ RE, 1.1 at both time points. The greatest increase in p-SAPK/JNK was seen with the combination of 3.1 μg mL^−1^ each of TE and RE in the CMT-12 cell line where densitometry values increased from 1.0 with DMSO treatment to 4.3 after 12 h incubation and 4.8 after 24 h incubation (*p* < 0.05 from DMSO treatment). Although there were similar increases in SAPK/JNK activation with TE and dual treatment of TE/RE (half doses) in C2 and D17 cell lines, these were not significantly different from DMSO control at 12 h or 24 h incubation time points. These results demonstrate possible mechanisms behind the observed susceptibility differences across the three cell lines, particularly in light of the heightened response of lesser doses of RE and TE in combination when compared to higher concentrations each extract independently in the CMT12 cell line.

## Discussion

Bioactive molecules derived directly from plants, or modeled after plant compounds, continue to be an active area of cancer research. The majority of these studies have been focused on human and rodent cancer models and the effects of these plant extracts and select compounds vary depending on species and cell origin [23, 24]. Few studies have been completed in dogs or related cell lines, therefore it is necessary to examine the effects of these compounds in vitro before using them in clinical veterinary trials. The aim of the current in vitro study was to examine the molecular effects of two natural extracts, turmeric root extract (rich in curcuminoids) and rosemary leaf extract (rich in carnosic acid), previously shown to inhibit proliferation synergistically in three established canine cancer cell lines [11]. These experiments were designed to focus on concentrations that may have utility in vivo and concentrations that showed synergistic effects of the compounds in our prior experiments (focusing on synergistic concentrations of 3 μg/mL of TE and RE versus 6 μg/mL^−1^ of each extract independently) [11]. In agreement with our previous proliferation and cytotoxicity results, cell treatment using TE alone was more potent than RE single treatment using the same extract concentrations and experimental conditions. TE had a greater effect on inducing cell apoptosis as measured by Caspase 3/7 activation and Annexin-V staining, and the combination treatment using only half the concentration of each extract induced a similar, if not greater, cell response. Varying degrees of susceptibility were detected across the three cancer cell lines used, with the CMT-12 cell line being the most susceptible to these treatments, perhaps due to a greater increase in intracellular curcumin accumulation as shown by flow cytometry. These differences across cell lines suggest the complexity in cellular response and potential susceptibility of various cell lines to treatment, further clarifying the need to understand what types of cancer may be more responsive to these interventions.

Prior studies have shown the autofluorescence of curcumin can be examined with flow cytometry [20]. An increase in curcumin fluorescence was seen across the three cell lines with the greatest signal measured in the CMT-12 cell line, especially when the two extracts were used in combination. A previous study in the human breast cancer cell line, MCF-7, showed an increase in intracellular accumulation of various chemotherapeutic drugs which was attributed to competitive inhibition of transmembrane transport pump P-glycoprotein by rosemary extract [25]. As this was not within the scope of our investigation, further experiments examining P-glycoprotein inhibition or curcumin utilization of other channels to enter cells are warranted to better understand the mechanisms by which rosemary enhances the accumulation of curcumin within cells.

The global effects of both TE, RE, and the two in combination showed no appreciable alteration in cell cycle kinetics. Of the three cell lines used in our experiments, only the C2 and D17 cells could be examined as a single cell suspension for cell cycle dynamics, while the CMT-12 cells displayed an artificial accumulation of cells in the G_2_/M phase. This was attributed to cell clumping in this cell line due to the fixation method used and use of propidium iodide causing cell-doublets to be inappropriately represented as G_2_M phase. There were some mild alterations in cell cycle in the D17 and C2 cell lines showing decreases in the G_1_/G_0_ and increases in G_2_/M phases with RE and the combination treatment. There were minimal to no cell cycle changes with TE extract alone, therefore further examination of cell cycle pathway analysis was not pursued. Prior literature has shown that curcumin can have significant effects on cell cycle dynamics through the upregulation of cyclins or cyclin dependent kinase activity [26–28]. In this previous literature, there was complete loss or greater than 50% reduction or increase in various portions of the cell cycle warranting further examination of cellular pathways involved. Generally, these differences may be due to the lower concentrations utilized in our experiments compared to the prior studies.

In line with pathway disruption and cell cycle dynamics, TE and RE are often thought to be antioxidants, however curcumin has been shown to cause oxidative damage to DNA and induction of the DNA damage response pathways that are intimately involved in cell cycle alteration or apoptosis [29, 30]. Our assessment of antioxidant status after treatment unanimously indicated that the cells are under less oxidative stress after treatment with either TE or RE alone or in dual combination within 12 h of incubation. To further assess the oxidative status, western blot analysis for gamma-histone H2A.X phosphorylation status was assessed in the three cell lines with TE, RE or dual treatment showing no phosphorylation in DMSO control or treated cells. Gamma-histone H2A.X is a marker of DNA oxidation and initiation of repair and was not detected when compared to UV irradiation as a positive control for DNA damage (data not shown). Under our cell culture conditions and extract concentrations used, we could not elicit a pro-oxidative response from TE or RE in any cell line used. This anti-oxidant property has been thought to be involved in cell survival and possible resistance to chemotherapeutic intervention [31]. Our data at these concentrations only suggest pro-apoptotic responses, suggesting that the mechanisms are unlikely to rely on oxidative damage.

Further examination of the cellular effects into cell signaling pathways previously implicated in TE and RE treatment were performed [25–35]. Concentrations that appeared to be most synergistic at inhibiting proliferation from our prior publication [11] were used to observe enhanced or diminished signaling events over extended periods of time from 12 to 24 h that might provide insights into the modest apoptotic response. Apoptosis could be, in part, due to overlapping effects on various signaling pathways including SAPK/JNK, ERK 1/2, STAT3, FAK, Src, mTOR, and membrane permeability proteins Bcl-2 and Bax [36, 37]. Previous literature has shown a synergistic effect between these two extracts, specifically the cleavage of poly ADP-ribose polymerase and Caspase-8, −9, and −3 on human cell lines [17]. Though there is relatively little primary literature on canine cell lines, one study has shown that a curcumin analog effectively alters STAT phosphorylation and activation in canine osteosarcoma cells [38]. After screening several signaling pathways, a consistent increase in the phosphorylated, or active, form of SAPK/JNK was detected with no consistent alterations in any other pathways examined via western blotting. This pathway has been implicated in driving cells to apoptosis when faced with environmental stressors such as oxidative stress, inhibition of protein synthesis, changes in the cell-matrix interaction, or signaling from inflammatory cytokines [39–41]. Consistent with our results, studies have shown that the downstream effects of SAPK/JNK activation are both cell and context dependent: pathway activation can be either pro-apoptotic or pro-proliferative. [42, 43] Changes in activation of MAPK/ERK were not observed after treatment in any of the cell lines examined. In general, early, transient activation of JNK may lead to cell survival, while sustained activation can induce apoptosis and curcumin or rosemary extracts appear to be involved in this constitutive activation of SAPK/JNK [44–46].

Differences in treatment responses were observed in the three cell lines. Our results showed an increase in phosphorylated SAPK/JNK after 12 h and 24 h of treatment with TE alone. RE induced a significant increase in phosphorylation after 12 h and 24 h of treatment in the CMT-12 cell line, while in the C2 cell line this increase was only seen at 12 h and returned to baseline by 24 h. The dual combination treatment had the greatest effect in the CMT-12 cell line, resulting in phosphorylated SAPK/JNK at levels greater than either extract alone (even using twice the concentration). Only the CMT-12 cell line showed sustained activation of SAPK/JNK with the combination treatment which may be the underlying reason behind the increased susceptibility of this cell line. SAPK/JNK has been implicated as a therapeutic target in certain contexts and patterns of activation whereby constitutive activation appears to be beneficial towards a pro-apoptotic response in a variety of cell lines and animal models [47–49]. The transient nature of activated SAPK/JNK in the C2 and D17 cell lines lead us to believe this may be involved in the diminished proliferation, however other pathways may be involved in the induction of apoptosis in these cancer cell lines. This data further demonstrates that the cell line and context specific effects of these extracts are vastly different and other approaches are needed to completely understand the complex interaction of the pathways involved during apoptosis induction.

Although the results of these experiments are promising, the clinical utility is complex due to the absorption, transformation and elimination kinetics of these compounds in general. The use of highly bioavailable curcumin is currently being examined and has 10–15% bioavailability [50]. Carnosic acid bioavailability from rosemary extract, though good, has not been studied extensively; however there is rapid glucuronidation, methylation, and oxidation of carnosic acid and related molecules from rosemary extract, and the bioactivity of these modified derivatives are unknown at this time [51].

## Conclusions

The results of this study provides some insights into possible mechanisms by which TE and RE induce apoptosis across three canine neoplastic cell lines. Our results indicate that different tumor types are likely to have a differential response to such interventions. The enhanced susceptibility found in the CMT-12 mammary cancer cell line may be due to the increased accumulation of curcumin when the combination treatment was used. In addition, sustained activation and signaling through the SAPK/JNK pathway may play a role in this cell line’s increased sensitivity to apoptosis. The results of this study warrant further investigations into the pharmacodynamics and pharmacokinetics of these extracts in dogs when incorporated into feed to determine if clinical trials are feasible.

## Abbreviations

AKT: Protein kinase B
BAX: BCL2-Associated X Protein
BCL2: B-Cell CLL/Lymphoma 2
DMSO: Dimethyl sulfoxide
ERK: Extracellular related kinase
FAK: Focal adhesion kinase
FBS: Fetal bovine serum
GMF: Geometric mean fluorescence
JAK: Janus kinase
mTOR: Mammalian target of rapamycin
PBS: Phosphate buffered saline
RE: Rosemary extract
ROS: Reactive oxygen species
SAPK/JNK: Stress activated kinase/jun-N-terminal kinase
STAT: Signal transducer and activator of transcription
TBST: Tris buffered saline tween
TE: Turmeric extract
